# Molecular Hydrogen in Drinking Water Protects against Neurodegenerative Changes Induced by Traumatic Brain Injury

**DOI:** 10.1371/journal.pone.0108034

**Published:** 2014-09-24

**Authors:** Kenji Dohi, Brian C. Kraemer, Michelle A. Erickson, Pamela J. McMillan, Andrej Kovac, Zuzana Flachbartova, Kim M. Hansen, Gul N. Shah, Nader Sheibani, Therese Salameh, William A. Banks

**Affiliations:** 1 Geriatric Research Education and Clinical Center, Veterans Affairs Puget Sound Health Care System, Seattle, WA, United States of America; 2 Department of Emergency Medicine, The Jikei University School of Medicine, Tokyo, Japan; 3 Division of Gerontology and Geriatric Medicine, Department of Medicine, University of Washington, Seattle, WA, United States of America; 4 Mental Illness Research Education and Clinical Center, Veterans Affairs Puget Sound Health Care System, Seattle, WA, United States of America; 5 Department of Psychiatry and Behavioral Sciences, University of Washington, Seattle, WA, United States of America; 6 Laboratory of Biomedical Microbiology and Immunology, Department of Microbiology and Immunology, University of Veterinary Medicine and Pharmacy, Kosice, Slovakia; 7 Division of Endocrinology, Department of Internal Medicine, Saint Louis University School of Medicine, Edward A. Doisy Research Center, St. Louis, MO, United States of America; 8 Opthalmology and Visual Sciences, University of Wisconsin School of Medicine and Public Health, Madison, WI, United States of America; Boston University School of Medicine, United States of America

## Abstract

Traumatic brain injury (TBI) in its various forms has emerged as a major problem for modern society. Acute TBI can transform into a chronic condition and be a risk factor for neurodegenerative diseases such as Alzheimer’s and Parkinson’s diseases, probably through induction of oxidative stress and neuroinflammation. Here, we examined the ability of the antioxidant molecular hydrogen given in drinking water (molecular hydrogen water; mHW) to alter the acute changes induced by controlled cortical impact (CCI), a commonly used experimental model of TBI. We found that mHW reversed CCI-induced edema by about half, completely blocked pathological tau expression, accentuated an early increase seen in several cytokines but attenuated that increase by day 7, reversed changes seen in the protein levels of aquaporin-4, HIF-1, MMP-2, and MMP-9, but not for amyloid beta peptide 1–40 or 1–42. Treatment with mHW also reversed the increase seen 4 h after CCI in gene expression related to oxidation/carbohydrate metabolism, cytokine release, leukocyte or cell migration, cytokine transport, ATP and nucleotide binding. Finally, we found that mHW preserved or increased ATP levels and propose a new mechanism for mHW, that of ATP production through the Jagendorf reaction. These results show that molecular hydrogen given in drinking water reverses many of the sequelae of CCI and suggests that it could be an easily administered, highly effective treatment for TBI.

## Introduction

Traumatic brain injury (TBI) has emerged as a signature injury of the early 21st century. In addition to blast injuries in theaters of war, TBI in the civilian population takes many forms with falls recently supplanting automobile accidents as the most common cause of TBI [1], [2]. Injuries that penetrate the skull are more rare but a very serious form of TBI [3]. Although TBI arises from a variety of injuries, the clincial endpoints that result and the mechanisms that drive the CNS towards those endpoints are thought to be shared not only among TBI’s, but also by a host of neurodegenerative diseases [4], [5]. These endpoints and mechanisms include brain edema, tauopathy, blood-brain barrier (BBB) disruption and dysfunction, and neuroinflammation [6], [7], [8]. Once set in motion, these processes often become self-reinforcing unless the cycle can be disrupted. Thus, preventative and interventive therapeutics are needed to treat TBI as early as possible.

Ideally, these treatments could be given immediately, even at the time of injury. This would require the therapeutic to have low toxicity and to be easily administered. One such potential therapeutic is molecular hydrogen. First introduced by Ohsawa et al in 2007 [9], molecular hydrogen acts at least in part as an anti-oxidant, combining with hydroxyl ions that are produced by CNS injuries, although other mechanisms may also exist [9]. As a low molecular weight (2 Da), uncharged molecule, molecular hydrogen has the ability to penetrate biological membranes. The membranes penetrated would include those that form the BBB; thus, molecular hydrogen should have unrestricted access to the CNS, a characteristic shared with few potential therapeutics. This high membrane penetrability explains why molecular hydrogen has therapeutic effects when given by a variety of routes. Here, we investigated the effects of molecular hydrogen given in drinking water (mHW), a method of delivery that should be readily translatable to clinical situations, on the brain edema, tau pathology, neuroinflammation, and gene expression induced by controlled cortical impact (CCI), an animal model of TBI.

The CCI model of TBI we used here is one of the gold standard models and delivers a controlled, reproducible, and quantified amount of mechanical force to the brain. According to the classification of Cernak [3], it is a dynamic, direct impact, penetrating injury model that results in direct contusion of brain tissue. It reproduces many of the characteristics of TBI seen in patients, including brain edema, alterations in cerebral blood flow, and metabolic changes and is widely used both in the investigations of mechanisms of TBI as well as for drug development [4], [10].

## Materials and Methods

### CCI Model

A stereotactic impactor (Leica Microsystems, Inc) was used to induce CCI in 8 week old C57BL/6 male mice (Harlan Sprague Dawley). All studies were conducted under protocols approved by the local animal care and use committee at the Veterans Affairs Puget Sound Institutional Animal Care and Use Committee, an AAALAC approved facility, and all studies adhered to the NIH Guide for the Care and use of Laboratory Animals. Anesthesia was induced with isoflurane (2–4% at 0.5 l/min) and maintained by administration with a nose cone. The head was shaved, the skin overlying the skull infiltrated with a lidocaine/bupivicaine mixture, and the mouse mounted in a stereotaxic apparatus that is part of a Lighthall stroke-contained pneumatic compression device. Using sterile technique, the skull was exposed through a midline incision and skin and muscle retracted. A 4 mm diameter opening was made in the skull 3 mm lateral and 2 mm caudal of the bregma, thereby exposing the right parietotemporal cortex. The bone was removed and the dura cut to expose the cortex. The cortex was compressed by using the impactor device to deliver a 3 mm diameter flat tip weight at a velocity of 5.82 m/s to a depth of 1.2 mm for a duration of 47 ms at a driving pressure of 73 psi. The injured area was covered with a 4 mm diameter artificial dura (GORE Peclude, WL Gore & Associates, Newark, NY) and a 5 mm diameter artificial bone plate made from dental cement (GC Fuji I, GC Corporation, Tokyo, Japan), the skin placed back into position and cemented, the isoflurane discontinued, and the mouse placed in an incubator at 37°C until regaining consciousness (return of righting reflex and mobility). Sham mice went through the entire procedure except for delivery of impact.

### Production and Administration of mHW

The mHW was prepared by dissolving molecular hydrogen (H2) in water under high pressure (1.6–1.8 ppm) to a supersaturated level using a hydrogen-water producing apparatus (Arega Co, Aich Japan). The saturated mHW was kept in an aluminum bag until use and freshly prepared each week and only water with a minimum hydrogen content of 1.6 mM was used. Mice were placed on mHW as their sole source of drinking water from 24 h before CCI/sham surgery until study. The mHW drinking water was given in glass water bottles having a stainless steel ball bearing; the ball bearing prevents degassing of the molecular hydrogen. We found that drinking, feeding, and grooming patterns did not differ between CCI and sham mice and that neither onset of drinking nor amount of water drank in 24 h was altered by CCI (data not shown).

### Brain Edema

Brains were harvested 24 h after sham surgery or 24 h, 3 days (72 h) or 7 days after CCI (n = 4–5/group). The hemi-cortex ipsilateral to CCI or sham surgery was weighed (wet weight), put in metal canisters that were placed on a heating plate at 125°C for 24 h, reweighed (dry weight), and the percent of water determined by the equation:
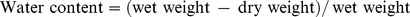



Additionally, the Edema Index of Keep et al [11] was applied to the 24 h time point using the equation:
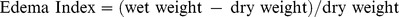



### Immunohistochemistry

Mice were examined by immunohistochemistry and the images presented are representative of each group. To prepare tissue for analysis, mice were anesthetized and fixed by transcardial perfusion with 4% paraformaldehyde. Brains were removed, paraffin embedded, and coronal sections were cut at 10 µm thickness and stored at 4°C until use. Sections were immunostained with AT8 (1∶250), Alz50 (1∶25), GFAP (1∶400), and IBA1 (1∶1000). Sections were deparaffinized and rehydrated through alcohols, and an antigen retrieval step consisting of heat pretreatment by microwave or autoclave in DakoCytomation Target Retrieval Solution (Vector, Burlingame, CA) was used. Sections were treated for endogenous peroxidases with 3% hydrogen peroxide in PBS (pH 7.4), blocked in 5% non-fat milk in PBS, incubated with primary antibody overnight at 4°C followed by biotinylated secondary antibody for 45 minutes at room temperature. Finally, sections were incubated in an avidin-biotin complex (Vector’s Vectastain Elite ABC kit, Burlingame, CA) and the reaction product was visualized with 0.05% diaminobenzidine (DAB)/0.01% hydrogen peroxide in PBS. Negative controls with secondary antibody alone did not immunostain tissue sections (data not shown). Photomicrographs were taken with a digital camera and imported into Adobe Photoshop for mounting. To optimize visualization of staining, photomicrographs were modified, when necessary, by adjusting brightness and contrast.

### mRNA Studies

Mice were divided into 3 groups of 6 each. Group 1 underwent sham surgery, group 2 underwent CCI, and group 3 underwent CCI but was started on mHW 24 h prior to CCI. All mice were sacrficed 4 h after CCI/sham surgery, and the hemicortex ipsilateral to surgery collected. The RNA was extracted from two pooled hemicortices using the MN nucleopsin II kit (Macherey-Nagel Inc, Bethlehem, PA). mRNA-seq libraries were constructed using the ScriptMiner kit (Epicentre, Madison, SI) and sequenced with TruSeq v3 chemistry on a HiSeq 2500 (Illumina, San Diego, Ca). Data was analyzed with GeneSifters software filtering tools (Geospiza, Seattle, WA).

### Brain Homogenate

The hemibrain ipsilateral to CCI/sham surgery was homogenized in 1 ml of extraction buffer (0.01 M phosphate buffered saline (PBS; 2.7 mM potassium chloride, 0.137 M sodium chloride, pH 7.4) 2.7 mM, 1 mM EDTA, 1 mM PMSF, Protease Inhibitor cocktail) using a mini beadbeater set at 4800 RPM for 30 sec to produce the PBS homogenate. Triton X-100 was added to the PBS homogenate to a final concentration of 0.1%, and vortexed vigorously. The homogenate was then centrifuged at 20,000 g for 10 min at 4°C. The resulting supernatant was used to measure brain cytokines and amyloid beta peptide (Aβ). Triton X-100 was added to a third portion of PBS homogenate to a final concentration of 1%, vortexed vigorously, and centrifuged at 20,000 g for 10 min at 4°C. The protein level of the resulting supernatant was measured (BCA, Pierce, Rockford, IL) and adjusted to 4 mg/ml with extraction buffer containing 1% Triton X-100 and frozen at −80°C as the 1% Triton X-100 extraction. Aliquots of this extraction were frozen and used for all Westerns and dot blots except for pan-tau immunoblotting.

For total tau detection, mouse brains were dissected from euthanized animals and rapidly snap frozen in liquid nitrogen prior to extraction. Mouse brain hemispheres were homogenized in high salt re-assembly buffer (RAB-High Salt [0.1 M MES, 1 mM EGTA, 0.5 mM MgSO¬4, 0.75 M NaCl, 0.02 M NaF, 0.5 mM PMSF, 0.1% protease inhibitor cocktail, pH 7.0]). For semi-quantitative immunoblotting, we detected mouse and human tau using the pan-tau antibody 17025 (provided as a generous gift of Virginia Lee, see [12] for details) at a dilution of 1∶3000 as described previously [13] and anti-actin antibody at 1∶1000 (DSHB). Rabbit polyclonal 17025 is a pan-tau antibody recognizing total mouse and human tau raised against full length recombinant tau.

### Serum and Brain Cytokine Measurements

At 4 h, 24 h, and 7 days after CCI or sham surgery, mice were anesthetized with urethane, the carotid artery exposed, severed, and blood collected from it, and the hemibrain ipsilateral to CCI/sham surgery was obtained and processed as above; the 0.1% Triton X-100 extract was used to measure brain cytokines. The arterial blood was centrifuged at 4500 g for 10 min at 4°C and cytokine levels measured on the resulting serum. Brain cytokine levels were measured at the 4 h, 24 h, and 7 day time points and serum cytokines on the 24 h and 7 day time points. The multi-plex kit for murine cytokines from BioRad was used and measures 23 cytokines: IL-1α, IL-1β, IL-2, IL-3, IL-4, IL-5, IL-6, IL-9, IL-10, IL-12(p40), IL-12(p70), IL-13, IL-17, Eotaxin (CCL11), G-CSF, GM-CSF, IFN-γ, KC (CXCL1), MCP-1 (CCL2), MIP-1α (CCL3), MIP-1β (CCL4), RANTES (CCL5), and TNF-α. Brain levels were reported relative to the protein content of the brain sample and are reported as pg/mg.

### Dot blots for Cyclophilin A and Amyloid Precursor Protein (APP)

Brains extracted in 1% Triton X-100 were analyzed for APP and Cyclophilin A expression by dot blot as described previously [14]. Briefly, 2 ug of protein in a volume of 250 ul PBS was loaded in duplicate wells of a Bio-dot microfiltration apparatus (Biorad). To normalize for antibody sensitivity, a standard curve was also prepared using one of the control samples with protein amounts per well ranging from 0.125 to 4 ug in a volume of 250 ul PBS and loaded in duplicate. Samples and standards were bound to a nitrocellulose membrane by vacuum, the membrane was washed, blocked in 5% milk, and incubated overnight with APP antibody (Epitomics, 1 µg/ml) or CypA antibody (Epitomics, 1 µg/ml) in blocking buffer. The membrane was then washed in PBS, and anti-rabbit secondary (Santa Cruz) prepared 1∶5000 in 5% milk and incubated with the membranes for 1 hour at room temperature. After washing, signal was visualized with West Pico chemiluminescent substrate. Images were captured using an ImageQuant LAS4000, and IQTL software used to quantify spot intensities. A standard curve was generated using spot intensities of the standards with Graphpad Prism software, and the spot intensity of each sample was normalized to the standard curve prior to statistical analysis.

### Aβ Assays

Sandwich ELISA kits which detect rodent Aβ40 or Aβ42 (Wako) were used to quantify Aβ levels. 0.1% Triton X-100 extracts were diluted 1∶1 in standard diluent provided in the kit, and subsequently assayed according to kit instructions. We have found that these kits specifically detect rodent Aβ40 and Aβ42 as evidenced by absence of signal for both analytes in APP knockout brains processed and assayed identically. Furthermore, this kit has been used by other groups to quantify changes in rodent Aβ in aging [15] and blast injury [16]. In the latter study, it was reported that the majority of rodent Aβ detected by this assay is found in the Triton-X fraction [16].

### Brain Homogenates for Aquaporin-4 (AQP-4), Hypoxia-inducible factor-1 alpha (HIF-1), Matrix Metalloproteinase (MMP)-2 & -9

The hemibrain ipsilateral to CCI/sham surgery was homogenized and extracted in Tris-EDTA extraction buffer (20 mM TRIS, 150 mM NaCl, 2 mM EDTA, 2% Triton X-100) with protease inhibitors. The brain extracts were run on a 4–12% Bis-Tris gels and transferred onto nitrocellulose membranes (Invitrogen, USA). The membranes were probed with a AQP-4 (Abcam, USA), HIF-1α (Novus Biologicals, USA), MMP-2 (R&D systems, USA) and MMP-9 (R&D systems, USA) primary antibodies, followed by horseradish peroxidase-conjugated secondary antibodies (Santa Cruz, USA). To verify uniformity of protein loading membranes were stripped and reprobed with GAPDH or β-actin antibody (Cell Signaling Technology, USA). The enhanced chemiluminescence western blot was digitalized with a LAS4000 CCD imaging system (GE Healthcare, USA) and analyzed by ImageQuant TL software.

### Brain-to-blood Efflux of Aβ and CSF Reabsorption

Brain-to-blood efflux of radioactively labeled Aβ and albumin were measured as previously described [17]. Murine Aβ_1–40_ was radioactively labeled with ^125^I and human serum albumin with ^131^I by the chloramine-T method and purified on columns of Sephadex G-10. Mice received an injection of 25,000 cpm of I-Aβ and 25,000 cpm of I-Albumin into the lateral ventricle of the brain contralateral to CCI and 10 min later the brain was harvested. Mice that had been dead between 10–30 min were used to estimate the amount of radioactive ligand available for transport. The amount of radioactive ligand transported was calculated as the difference between the 10 min value in alive mice and the 10 min value in dead mice.

### Effects of mHW on Cellular Respiration and ATP Production

Conditionally immortalized mouse cerebral pericyte cells (ImBPC) established as previously described [18] were cultured in growth medium, which consisted of 5.5 mM glucose Dulbecco minimal essential media (DMEM) (Sigma-Aldrich, St. Louis, MO) supplemented with 10% fetal bovine serum (FBS), non-essential amino acids (NEAA), interferon-γ (44 U/ml; R&D Systems, Minneapolis, MN), penicillin (100 U/mL), and streptomycin (0.1 mg/mL). All cultures were maintained at 33°C under 5% CO_2_. For pretreated cell experiments, media was freshly prepared using mHW (1.6 mM) or deionized water with Dulbecco minimal essential media powder (DMEM) (Sigma-Aldrich, St. Louis, MO) supplemented with 10% fetal bovine serum (FBS), non-essential amino acids (NEAA), interferon-γ (44 U/ml; R&D Systems, Minneapolis, MN), penicillin (100 U/mL) and streptomycin (0.1 mg/mL). Cells for Seahorse analyzer experiments were harvested using 1× Trypsin containing EDTA (Sigma-Aldrich, St. Louis, MO) and plated into 24-well culture plates at a density of 5×10^4^ cells/well. An n = 10 was used in each experiment and each experiment was completed at least three times. Cells for ATP assay were harvested and plated into 96-well culture plates at a density of 5×10^3^ cells/well. An n = 8 was used in each experiment and each experiment was completed three times.

A Seahorse Bioscience XF24 Extracellular Flux Analyzer (Seahorse Bioscience, North Billerica, MA) was used to measure the rate of oxidative metabolism of glucose (respiration). Under typical in vitro cell culture conditions, the rate of oxygen consumption (OCR) is an indicator of mitochondrial respiration and the extracellular acidification rate (ECAR) is predominantly a measure of lactic acid formed during glycolytic energy metabolism. ImBPC were seeded into a specialized Seahorse microplate (V7; 0.32 cm^2^ growth area) at 5×10^4^ cells per well in 100 µl of growth medium and incubated at 33°C in 5% CO_2_ for 2 h. An additional 150 µl of medium was added after the cells had adhered. On the following day, assays were initiated by replacing the media with XF-DMEM (supplemented with 5 mM glucose and 1 mM sodium pyruvate at pH 7.4) nonbuffered media and incubating at 37°C for 60 min to allow the temperature and pH to reach equilibrium. The microplate was then placed into the XF24 instrument to measure OCR and ECAR. Basal measurements of OCR and ECAR were established, and inhibitors of mitochondrial respiration were injected sequentially as indicated to determine mitochondrial function and glycolysis. The inhibitors were used at the following concentrations: oligomycin- 3 µM; carbonyl cyanide 4-(trifluoromethoxy)phenylhydrazone (FCCP)- 3 µM; rotenone- 3 µM; and antimycin A- 1.5 µM. ATP production is measured as (basal respiration – oligomycin respiration). Proton leak is measured as (oligomycin respiration – rotenone/Antimycin A respiration). Maximal respiration is the respiration measured after FCCP treatment. Reserve capacity is measured as (FCCP respiration – basal respiration). Non-mitochondrial respiration is the respiration that occurs after rotenone/Antimycin A treatment.

ATP levels in ImBPC were measured using an ATPlite detection assay (Perkin Elmer; Waltham, MA) according to manufacturer’s protocol. Briefly, cells were lysed at various time points in mammalian cell lysis solution followed by incubation in substrate solution. After 10 min for dark adaption, luminescence was measured using the Victor^3^ multi label plate reader (Perkin-Elmer; Waltham, MA).

### Statistical Analysis

Results are reported at the mean +/− the standard error of the mean. Two means were compared by two-tailed t-test. More than two means were compared by analysis of variance (ANOVA) followed by Newman-Keuls post-test. Studies done using the XF24 Seahorse Analyzer were analyzed using AUC ANOVA software provide by Seahorse Bioscience.

## Results

### Brain Edema

**Figure 1:**
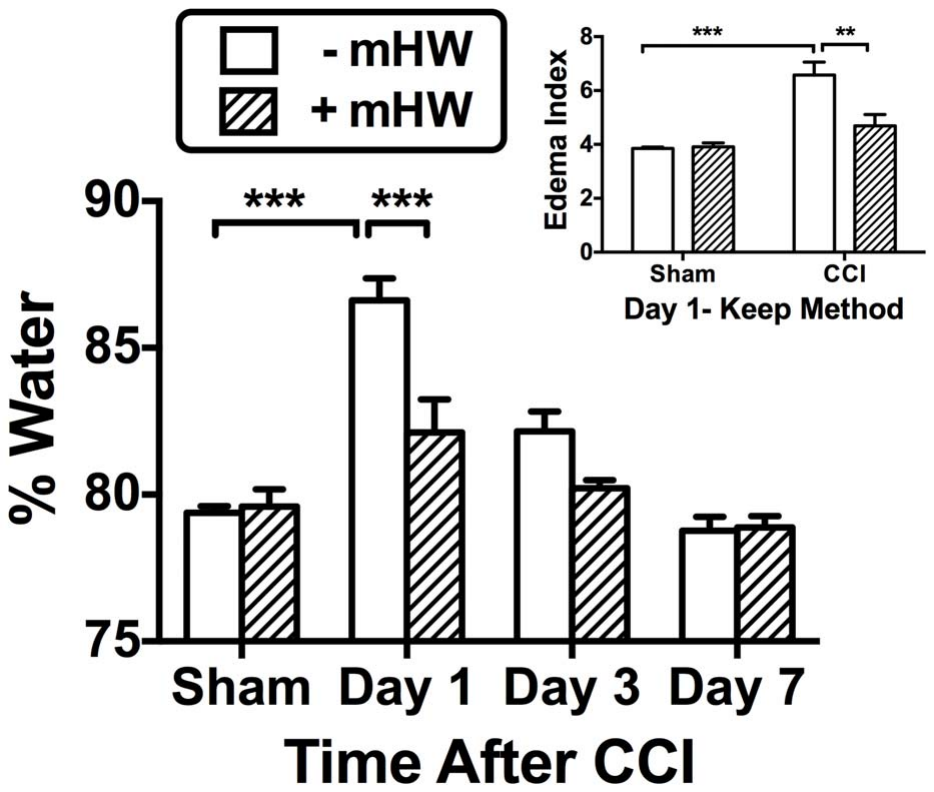
Effects of CCI and mHW on Brain Edema. Main figure shows that the percent of brain weight that was water increased 24 h (day 1) after CCI and that mHW significantly reduced the water content. Inset shows brain edema index calculated by the Keep method at 24 h; CCI increased the edema index, while mHW was protective. **p<0.01, ***p<0.001.

Figure 1 shows the results of CCI and treatment with mHW on the percent of water content in the ipsilateral hemi-brain. A two way ANOVA showed statistical significance for day [F(3,30) = 30.4, p<<0.001], treatment [F(1,30) = 11.2, p<0.01], and interaction [F(1,30) = 5.9, p<0.01] for % water. Tukey‘s post-test showed that % water was significantly increased 24 h after CCI (p<0.001), but was inhibited to a statistically significant degree by mHW (p<0.001). Analysis of the 24 h data using the Keep method [11] showed significant edema that was reversed by mHW (inset).

### Immunohistochemistry & Immunoblotting

**Figure 2:**
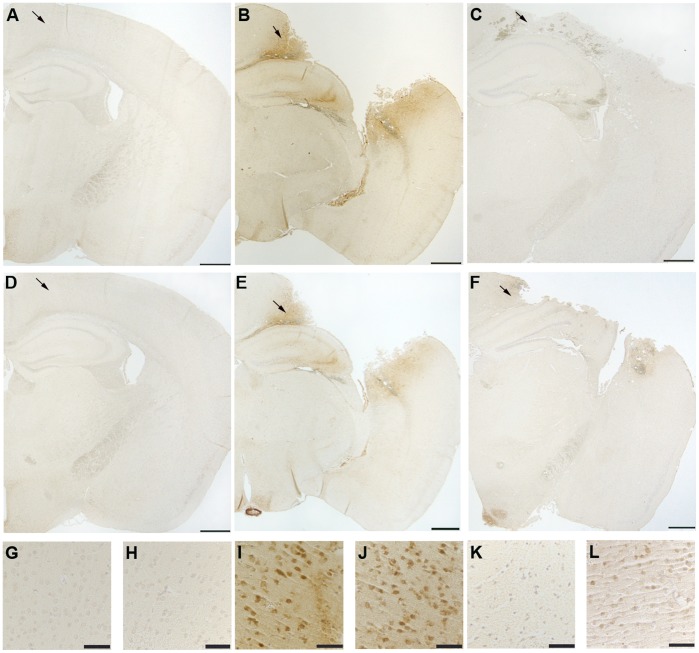
Reduction of CCI-induced pathological tau expression in hippocampus and cortex by mHW. The CCI-induced increase in AT8 and Alz50 immunoreactivity is attenuated by treatment with hydrogen water. Representative images of immunostaining in brains from sham (A, D, G, H), CCI (B, E, I, J), and CCI+mHW treated (C, F, K, L) animals. Low power representative images depict AT8 (A–C) and Alz50 (D–F) immunoreactivity. G–L, high power images of the cortical region designated by arrows, immunostained for AT8 (G,I,K) and Alz50 (H, J, L). Scale bars: 500 µm, A–F; 50 µm, G–L.

**Figure 3:**
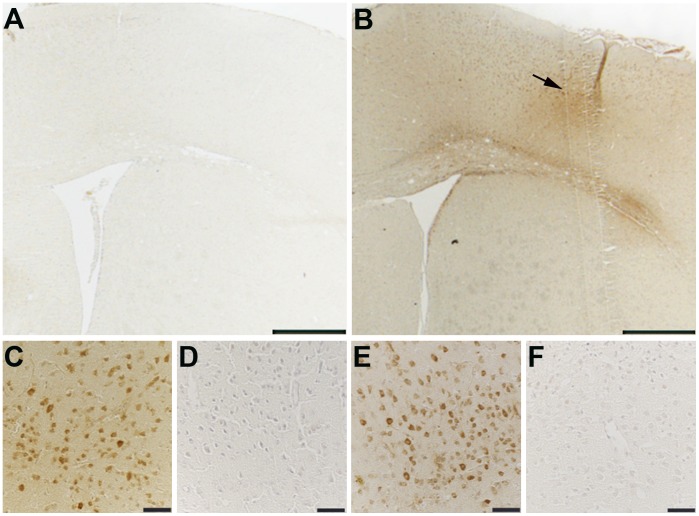
Reduction of CCI-induced pathological tau by mHW in frontal cortex. CCI induced increases in AT8 and Alz50 immunoreactivity extend to the frontal cortex and are attenuated by treatment with hydrogen water. Low power representative images demonstrate increased AT8 immunoreactivity in the frontal cortex of CCI (B) compared to sham treated (A) mice. C–F, high power images of the cortical region shown in B (arrow) demonstrate AT8 (C,D) and Alz50 (E,F) immunoreactivity in CCI (C,E) and HW treated (D,F) mice. Scale bars: 500 µm, A,B; 50 µm, D–F.

**Figure 4:**
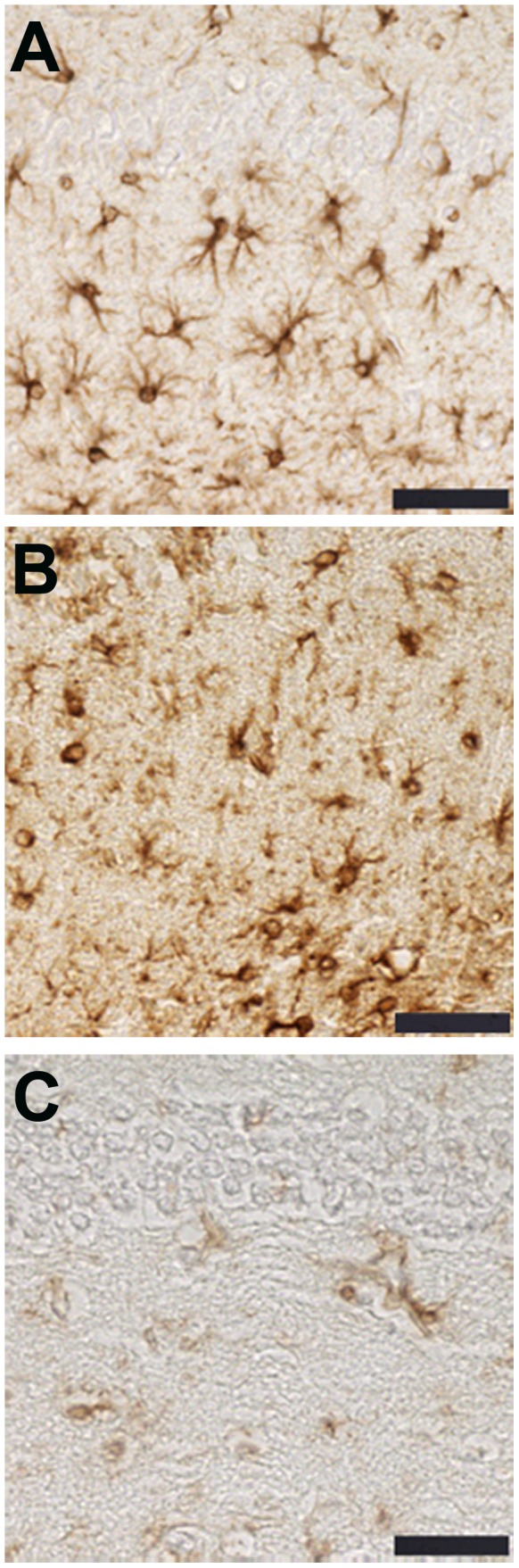
GFAP immunoreactivity is decreased in the hippocampus (CA1 stratum radiatum) of CCI mice treated with hydrogen water. Representative images demonstrate similar levels of hippocampal GFAP immunoreactivity in sham (A) and CCI (B) mice. Immunostaining for GFAP is greatly reduced in the hippocampus of animals treated with hydrogen water (C). Scale bars: 50 µm.

**Figure 5:**
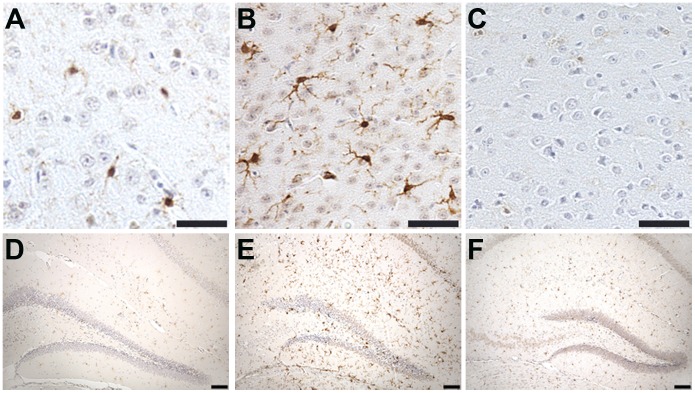
The CCI induced increase in Iba1 immunoreactivity is attenuated by treatment with hydrogen water. Representative images are of Iba1 immunoreactivity in the cortex adjacent to the site of injury (A–C) and the hippocampus (D–F) of sham (A,D), CCI (B,E) and CCI/HW treated (C,F) animals. Scale bars: 50 µm, A–C; 100 µm, D–F.

Staining of phosphorylated tau with both AT8 and Alz 50 were greatly increased in the cortex of CCI mice (Figures 2 & 3) with extension into the hippocampal CA3 region (Figure 2). Total tau as detected with the pan-tau antibody 17026 was not altered 12, 18, 24, or 48 h after CCI (data not shown). Treatment with mHW abolished these increases in tau (Figures 2 & 3). CCI did not produce a remarkable increase in GFAP staining, but CCI mice treated with mHW had less GFAP staining that either sham or CCI mice (Figure 4). CCI increased Iba1 immunostaining; mHW decreased Iba1 immunostaining to levels lower than those seen in sham mice. (Figure 5).

### Serum and Brain Cytokine Measurements

**Figure 6:**
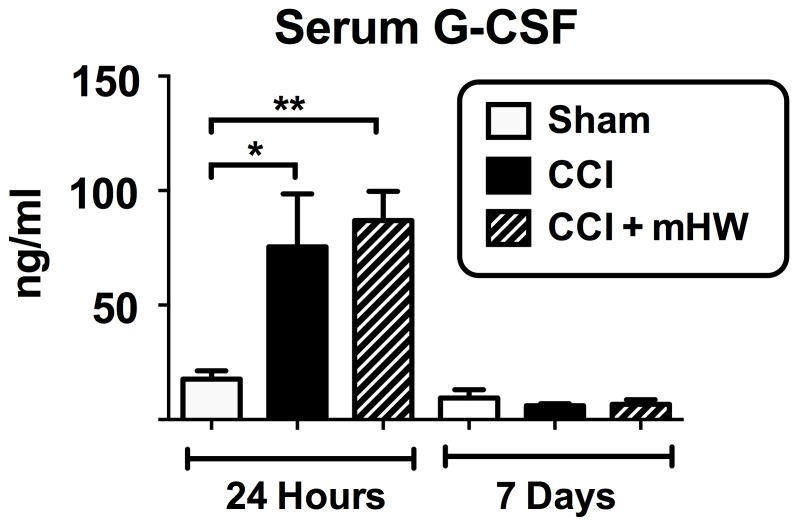
Effects of CCI and mHW on Serum Cytokine Levels. Only G-CSF was elevated in blood after CCI and only at 24 h. There was no statistically significant effect of mHW on serum G-CSF levels. *p<0.05; **p<0.01.

For serum, only granulocyte-colony stimulating factor (G-CSF) was significantly affected by CCI in comparison to sham surgery and only at 24 h (Figure 6); mHW had no effect.

**Figure 7:**
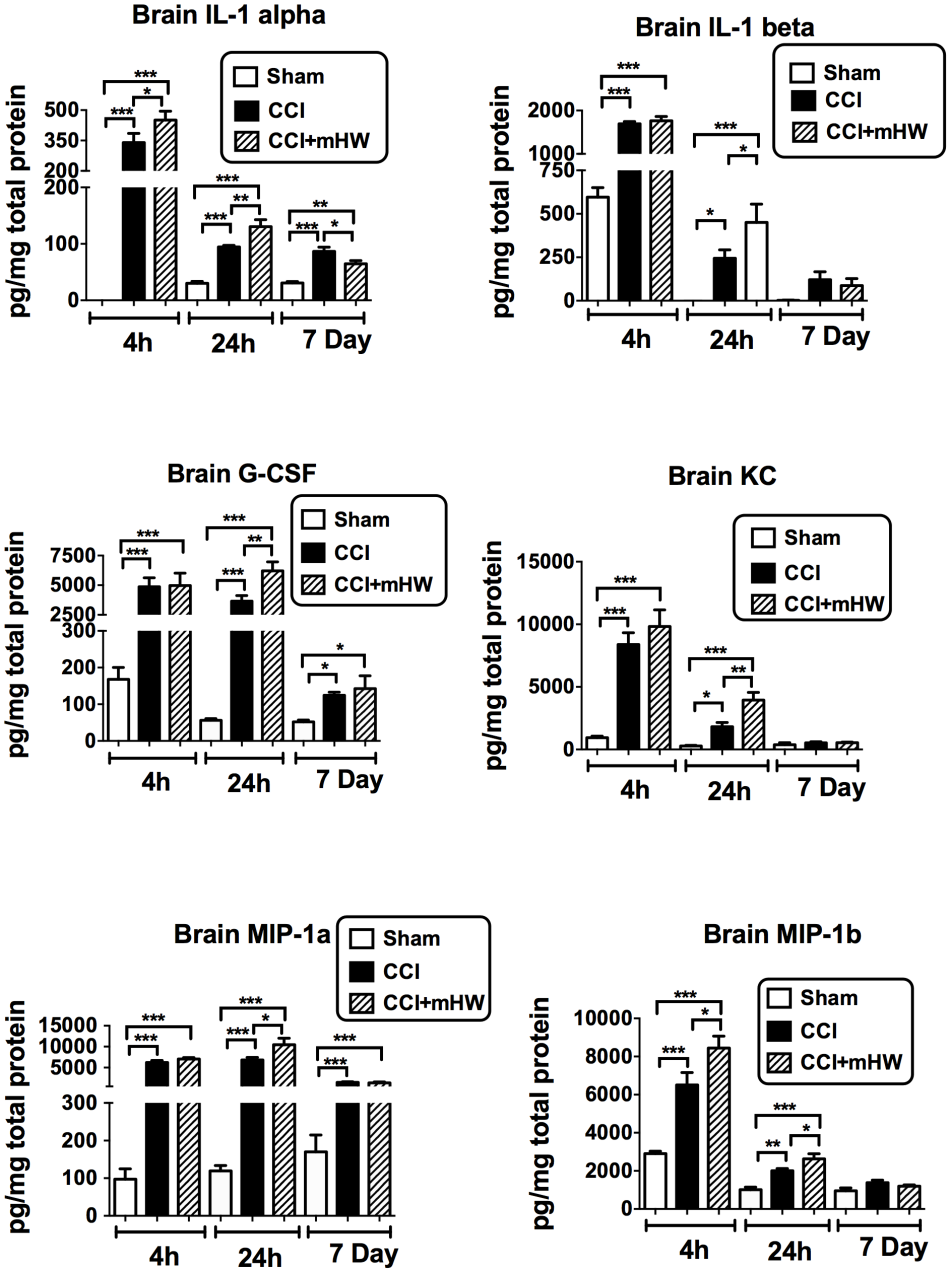
CCI-induced Elevations in Six Cytokines in Brain Were Affected by mHW. IL-6 (not shown; see Table 1 for results) had a pattern similar to G-CSF. Except for the day 7 IL-1α value, the statistically significant effects of mHW were to further increase brain cytokine levels. *p<0.05; **p<0.01; ***p<0.001.

Because of an apparrent effect of sham surgery on brain cytokine levels at 4 h, separate ANOVAs were conducted for the 4 h, 24 h, and 7 day results. Three cytokines were not detectable in the brain sample (IL-9, eotaxin, INF-gamma). Table 1 shows the effects of CCI and mHW on brain levels of the other 20 cytokines. Six of the detected cytokines were not affected by CCI (IL-2, IL-5, IL-10, IL-13, IL-17, TNF). Of the remaining 14 cytokines, all had elevated brain levels at one or more time points after CCI. Eight were elevated at all time points (IL-1α, IL-4, IL-6, IL-12p40, G-CSF, MCP-1, MIP-1α, RANTES), four at 4 h and 24 h but not at 7 days (IL-1β, IL-12p70, KC, MIP-1β), 1 at 4 h only (GM-CSF) and one at 24 h and 7 days but not at 4 h (IL-3). Seven cytokines had CCI+mHW levels at one or more time points that differed significantly from CCI levels (Table 1 & Figure 7). Except for the IL-1α 7 day value, the CCI+mHW values were higher than those for CCI alone. These increases were statistically significant at 4 h and 24 h for IL-1α and MIP-1α and at 4 h for IL-1β, G-CSF, KC, and MIP1α (Figure 7). For many cytokines, the CCI value tended to be higher at 7 days than the CCI+mHW value, but only reached statistical significance for IL-1α. TNF levels were lower in CCI+mHW than in sham mice 4 h after CCI and IL-13 levels were higher in CCI+mHW than in sham mice 24 h after CCI.

**Table 1:** Effect of CCI and CCI+mHW on Brain Cytokine Levels.

	4 h Post CCI			24 h Post CCI			7 Days Post CCI		
	Sham (n = 6)	CCI (n = 5–6)	CCI+mHW (n = 5)	Sham (n = 7)	CCI (n = 7)	CCI+mHW (n = 7)	Sham (n = 4)	CCI (n = 6)	CCI+mHW (n = 4)
IL-1a	0±0	339±46**	451±44**#	30±3	94±2**	131±12**##	31±2	86±8**	65±6**#
IL-1b	595±54	1692±53**	1766±97**	14±10	244±48**	451±105**#	2±2	121±45	87±40
IL-2	50±13	64±10	58±6	9±5	3±1	14±4	14±7	18±6	8±4
IL-3	65±4	65±3	61±6	26±2	53±3**	60±6**	27±2	39±3*	37±1*
IL-4	113±3	165±11**	178±8**	50±3	67±2**	77±6**	48±3	66±4**	60±1*
IL-5	138±7	153±6	157±12	87±5	90±4	91±7	89±5	98±5	87±6
IL-6	70±7	694±56*	1003±293**	25±3	575±101*	1155±271**#	22±2	36±3*	34±2*
IL-10	487±26	475±37	482±41	160±19	186±24	205±26	143±11	180±17	182±19
IL-12p40	847±31	1897±220**	2249±183**	281±23	698±47**	616±43**	307±35	803±92**	879±76**
IL-12p70	37±12	747±76**	868±75**	102±23	269±42*	293±54*	92±28	134±20	130±15
IL-13	2271±92	2129±145	2366±235	510±60	696±62	840±80**	512±41	475±31	454±41
IL-17	595±25	617±13	562±39	242±8	208±10	195±21	224±22	209±12	212±20
G-CSF	168±32	4864±762**	4974±1045**	56±4	3650±477**	6212±765**##	52±5	125±8*	142±35*
GM-CSF	587±73	1821±75**	1840±65**	17±17	221±80	232±134	60±60	0±0	0±0
KC	943±125	8390±938**	9826±1332**	264±52	1817±346*	3950±616**##	363±165	535±80	538±60
MCP-1	2715±382	10833±1305**	14079±1822**	966±76	13593±1264**	15835±1457**	1007±110	2908±327**	2516±342**
MIP-1a	97±28	6229±474**	7039±353**	119±15	6824±612**	10445±1586**#	170±44	1475±148**	1351±214**
MIP-1b	2904±125	6507±652**	8443±629**#	1004±132	2003±119**	2628±273**#	954±150	1377±133	1200±66
RANTES	63±3	110±14*	136±17**	38±5	160±11**	145±21**	73±19	262±28**	220±24**
TNFa	10130±511	8512±463	7868±828*	7259±778	6828±582	6243±578	9109±1909	7188±594	7209±698

*p<0.05 & **p<0.01 for CCI vs Sham and for CCI+mHW vs Sham; #p<0.05 & ## p<0.01 for CCI+mHW vs CCI.

Mean ± SE (pg/mg of brain protein).

### mRNA studies

After CCI, 246 genes had increased expression by 2 fold or more and 38 genes had expression that was decreased by 50% or more. mHW reduced by 50% or more 236 genes whose expression was increased by 2 fold or more by CCI. We used GeneSifter to analyze which categories of Biological Processes (Table 2), Cellular Components (Table 3), and Molecular Functions (Table 4) were most affected, restricting categories to those containing a minimum of 6 genes and having at least 3 genes altered. Z-score was used as a further filter with an absolute value of ≥3.5 for Biological Processes and ≥2.0 for Cellular Components and Molecular Functions. Of 15 significant categories for Biological Processes, all Z-scores indicated positive enrichment and recurring categories related to cytokine release (3 categories), oxidation/carbohydrate metabolism (4 categories), and leukocyte or cell migration/chemotaxis (3 categories). 8 categories involved transport/secretion with most overlapping with an above category (e.g., cytokine transport). These findings suggest that mHW is very effective at reversing CCI-activation of genes related to oxidation, carbohydrate metabolism, and neuroinflammation. Molecular Functions that were positively enriched included enzymatic genes, receptor genes, and transporter, channel, and transmembrane transporter genes. Molecular Functions was negatively enriched in genes relating to ATP and nucleotide binding. Cellular Components was positively enriched in those genes related to the extracellular and cell membrane environment and negatively enriched in those related to the intracellular, nuclear, and cytoplasmic environments.

**Table 2:** Changes in Brain Expression with CCI and mHW: Biological Processes.

Biological ProcessesPositive Z-score	# Affected Genes	# in Gene Set	Z-Score
Positive regulation of cytokine transport	3	33	5.21
Positive regulation of hormone secretion	4	61	4.91
Positive Regulation of Secretion	7	170	4.72
Carbohydrate Homeostasis	4	67	4.62
Glucose Homeostatis	4	67	4.62
Regulation of Oxidoreductase Activity	3	41	4.56
Leukocyte Chemostasis	4	74	4.32
Regulation of Cytokine Secretion	3	45	4.30
Leukocyte Migration	5	114	4.17
Feeding Behavior	4	83	3.99
Cytokine Secretion	3	52	3.91
Positive Regulation of Protein Secretion	3	53	3.86
Regulation of Secretion	9	9	3.86
Cell Chemotaxis	4	89	3.80
Secretion by Cell	11	469	3.63

Categories listed consisted of a minimum of 6 genes, had at least 3 genes that were changed, and a Z-score of >3.63.

**Table 3:** Changes in Brain Expression with CCI and mHW: Cellular Component.

Cellular ComponentPositive Z-score	# Affected Genes	# in Gene Set	Z-Score
Brush Border	3	62	3.60
Extracellular Region	27	1766	3.16
Cell-cell Junction	5	233	2.13
Negative Z-score			
Intracellular	59	10670	−4.79
Intracellular Part	59	10453	−4.52
Organelle	50	8945	−4.01
Intracellular Organelle	50	8919	−3.98
Nucleus	20	4806	−3.78
Membrane-bounded Organelle	45	7998	−3.62
Cell	105	14900	−3.61
Cell Part	105	14900	−3.61
Intracellular Membrane-bounded Organelle	45	7982	−3.60
Cytoplasm	45	7841	−3.43
Intracellular Organelle Part	16	3899	−3.36
Intracellular non-membrane-bounded Organelle	6	2328	−3.33
Non-membrane-bounded Organelle	6	2328	−3.33
Organelle Part	17	3978	−3.27
Macromolecular Complex	14	3198	−2.80
Cytoskeleton	3	1408	−2.72
Protein Complex	12	2670	−2.46
Cytosol	4	1421	−2.45
Cytoplasmic Part	33	5320	−2.19
Nuclear Part	7	1714	−2.11
Membrane-enclosed Lumen	7	1658	−2.11

Categories listed consisted of a minimum of 6 genes, had at least 3 genes that were changed, and a Z-score of ≥2.0.

**Table 4:** Changes in Brain Expression with CCI and mHW: Molecular Function.

Molecular FunctionPositive Z-score	# Affected Genes	# in Gene Set	Z-Score
Methyl Indole-3-acetate Esterase Activity	3	7	12.01
Methyl Jasmonate Esterase Activity	3	7	12.01
Methyl Salicylate Esterase Activity	3	7	12.01
Neuropeptide Receptor Binding	3	21	6.65
Hormone Activity	6	118	4.97
Carboxylesterase Activity	5	97	4.58
Serine Hydrolase Activity	7	203	4.00
G-protein-coupled Receptor Binding	6	183	3.55
Serine-type Peptidase Activity	6	200	3.28
Serine-type Endopeptidase Activity	5	155	3.19
Sodium Ion Transmembrane Transporter Activity	3	80	2.80
Cytokine Activity	5	183	2.75
Acyltransferase Activity	5	193	2.61
Transferace Activity, Transferring Acyl Groups Other Than Amino-acyl Groups	5	193	2.61
Transmembrane Transporter Activity	14	835	2.59
Channel Activity	8	388	2.58
Receptor Binding	16	999	2.58
Passive Transmembrane Transporter Activity	8	389	2.57
Transferase Activity, Transferring Acyl Groups	5	198	2.54
Transporter Activity	16	1026	2.47
Monovalent Inorganic Cation Transmembrane Transporter Activity	4	149	2.41
Ion Transmembrane Transporter Activity	11	654	2.30
Substrate-specific Transmembrane Transporter Activity	12	754	2.20
Substrate-specific Channel Activity	7	376	2.11
Substrate-specific Transporter Activity	13	863	2.09
Negative Z-score			
Adenyl Nucleotide Binding	4	1446	−2.49
Adenyl Ribonucleotide Binding	4	1439	−2.48
ATP Binding	4	1414	−2.44
Nucleotide Binding	9	2096	−2.26
Nucleic Acid Binding	12	2510	−2.22
Purine Ribonucleoside Triphosphate Binding	7	1718	−2.13
Binding	77	10525	−2.03

Categories listed consisted of a minimum of 6 genes, had at least 3 genes that were changed, and a Z-score of ≥2.0.

### AQP-4, HIF-1, and MMP-2 &- 9 Measures

**Figure 8:**
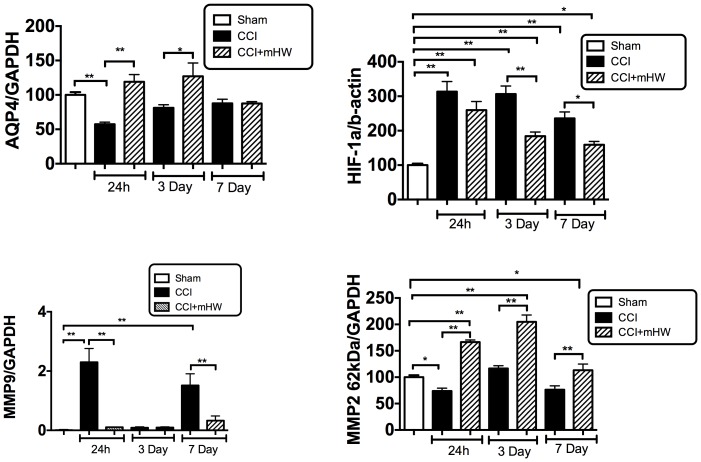
Effects of CCI and mHW on AQP-4, HIF-1, and MMP-2 & -9 Levels. The upper left panel shows that CCI suppressed AQP-4 expression at 24 h and that mHW blocked this decrease. HIF-1 (upper right panel) was increased at all times after CCI and mHW induced a partial recovery. MMP-9 (lower left panel) showed elevations 24 h and 7 days after CCI that were blocked by mHW. MMP-2 was decreased 24 h after CCI but mHW increased levels at all time points.

AQP-4 was suppressed 24 h after CCI but had recovered to baseline by 3 days (Figure 8, upper left panel). Treatment with mHW induced a statistically significant increase in comparison to CCI at 24 h and 3 days. HIF-1 was significantly increased by CCI at all time points, peaking at 24 h; treatment with mHW suppressed this increase to a statistically significant degree at day 3 and 7, although HIF-1 was still elevated in comparison to sham treated mice (Figure 8, upper right panel). MMP-9 showed a biphasic increase, being elevated at 24 h and 7 days but not at 3 days; mHW reversed these increases (Figure 8, lower left panel). MMP-2 decreased 24 h after CCI; mHW induced a statistically signifcant increase in MMP-2 at all time points in comparison both to the sham and to the time-matched CCI groups (Figure 8, lower right panel).

### Measures of Cyclophilin A, APP, and Aβ

**Figure 9:**
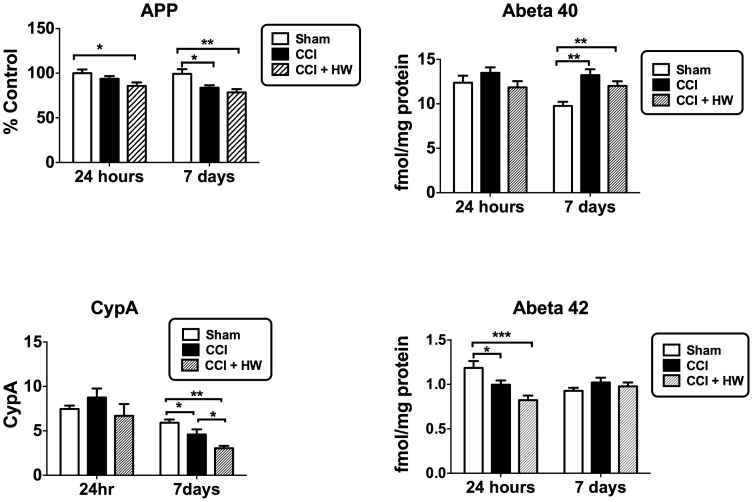
Effects of CCI and mHW on Cyclophillin A, APP, and Amyloid Beta Peptide Levels. The upper left panel shows that protein levels of APP were decreased 7 days after CCI and that mHW did not protect from CCI. Amyloid beta peptide_1–40_ (upper right panel) was increased on day 7 and amyloid beta peptide_1–42_ (lower right panel) was decreased 24 h after CCI, but mHW did not alter these effects of CCI. CypA was decreased 7 days after CCI and this decrease was enhanced by mHW (lower left panel).

CCI reduced CypA levels 7 days but not 24 h after CCI; mHW further reduced CypA at 7 days after CCI (Figure 9, lower left panel). Protein levels of APP were decreased by CCI at 7 days, but not at 24 h (Figure 9, upper left panel). The CCI+mHW group did not differ from the CCI group at either time point, but was lower than sham mice at both 24 h and 7 days. Aβ_1–40_ was not affected at 24 h but was increased at 7 days in both the CCI and CCI+mHW groups (Figure 9, upper right panel). Aβ_1–42_ was decreased 24 h after CCI and CCI+mHW but was not altered by 7 days; there were no differences at either time for CCI vs CCI+mHW (Figure 9, lower right panel). The Aβ_1–42_/Aβ_1–40_ ratios were lower at both 24 h and 7 days for both CCI and CCI+mHW groups, but showed no differences between CCI vs CCI+mHW (data not shown).

### CSF Reabsorption and Brain-to-blood Efflux of Aβ

Neither CSF reabsorption as measured by I-Albumin efflux nor Aβ efflux was affected 24 h, 3 days, or 7 days after CCI (data not shown).

### Effects on Cellular Respiration

**Figure 10:**
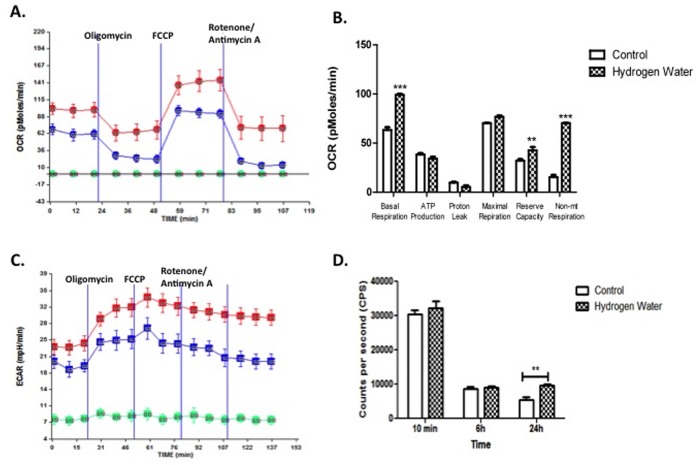
Pretreatment with Molecular Hydrogen Enhances Mitochondrial Aerobic Metabolism. ImBPCs were subjected to a mitochondrial stress test after treatment with molecular hydrogen water (red) or control (dI water; blue) for 24 h. Oxygen consumption rates (OCR) (Panel A) and extracellular acidification rates (ECAR) (Panel C) were measured using the Seahorse XF24 analyzer in the presence of the ATP synthase inhibitor (oligomycin; 3 µM), the uncoupling agent (FCCP; 3 µM), the complex I inhibitor (rotenone; 3 µM), and the complex III inhibitor (antimycin A; 1.5 µM). Analysis of OCR data showed molecular hydrogen increased basal respiration, reserve capacity, and non-mitochondrial (non-mt) respiration but had no effects on ATP production rate as assessed indirectly by OCR analysis, proton leak, or maximal respiration (Panel B). Direct measurement of ATP levels (Panel D) in other cells not exposed to the metabolic stressors showed higher levels of ATP after 24 h but not after 10 min or 6 h of exposure to molecular hydrogen. Results presented here are from one experiment completed, which is representative of the 3 experiments that were done. Results are represented as a mean ± SEM. ** is p<0.01 and *** is p<0.001.

To assess mitochondrial function that occurs in response to mHW, a Seahorse analyzer assay was performed by exposing the mHW-pretreated ImBPC to oligomycin, FCCP (carbonyl cyanide p-trifluoromethoxyphenylhydrazone) and rotenone/antimycin A in succession and measuring OCR (Figure 10A) and ECR (Figure 10C). The results showed that molecular hydrogen increased basal respiration, reserve capacity, and non-mitochondrial respiration, but not maximal respiration, proton leak, or the oxygen-dependent production of ATP (Figure 10B). The direct measure of ATP showed increased levels after 24 h of exposure to mHW, but not after a 10 min or 6 h exposure.

**Figure 11:**
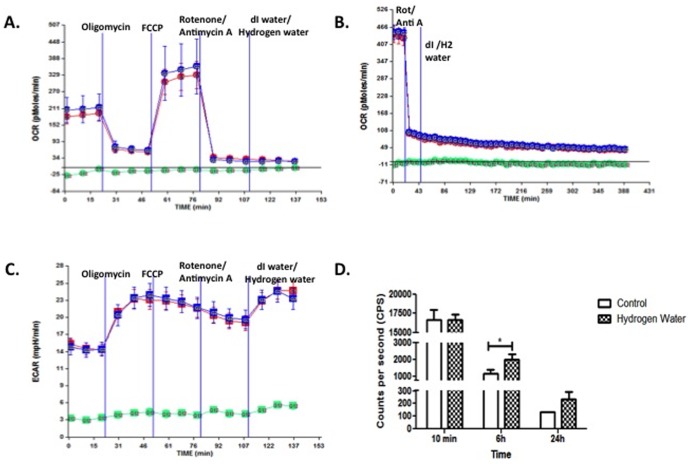
Treatment with Molecular Hydrogen Does Not Rescue Respiration After ETC Inhibition. ImBPCs were subjected to a mitochondrial stress test in the presence of the ATP synthase inhibitor (oligomycin; 3 µM), the uncoupling agent (FCCP; 3 µM), the complex I inhibitor (rotenone; 3 µM), and the complex III inhibitor (antimycin A; 1.5 µM). Once respiration was inhibited, ImBPCs were exposed to hydrogen water (red) or control (dI water; blue), and OCR was measured for 30 min (Panel A) or 6 h (Panel B). ECAR was also examined for 30 min (Panel C). ATP levels were measured after 10 min, 6 h, or 24 h of treatment with molecular hydrogen water. Results presented here are from one experiment completed, which is representative of the 3 experiments that were done. Results are represented as a mean ± SEM. ** is p<0.01 and *** is p<0.001.

In other ImBPCs (Figure 11), the ETC was shut off before exposure to molecular hydrogen. OCR and ECAR were unaffected by molecular hydrogen (Fig. 1 A–C) as measured for up to 6 h. However, molecular hydrogen was associated with an increase in ATP levels after 6 h of exposure (Figure 11D).

## Discussion

TBI results in long-term injuries to the CNS that involve immediate effects, such as brain edema, and more sustained effects, such as neuroinflammation. Here, we found that many of the immediate and sustained effects were modulated by treating mice with mHW.

Edema is a significant clinical end point in the pathophysiology of TBI. It occurs because of ongoing events, including BBB disruption, and it leads to further damage to the brain from multiple mechanisms that can include pressure, electrolyte disturbances, and the toxins from BBB leakage. Edema peaked at our 24 h point with the arithmetic increase seen at the third day (72 h after CCI) not being statistically significant. mHW decreased CCI-induced edema by about half at 24 h. Edema was also reduced as measured by the Keep method. As the Keep method uses dry weight as the denominator, it is much less influenced by either edema or tissue loss than the traditional method that uses wet weight in the denominator. The results showed that water content increased from being 4-fold greater than dry weight to 6.5-fold greater with CCI and that mHW preserved the ratio at 4.7-fold.

TBI, stroke, and hypoxia alter the expressions and functions of AQP-4, HIF-1, and the MMP’s; these in turn affect edema formation, BBB disruption, and alterations in brain interstitial fluid circulations [19]. Cyclophilin A is associated with BBB disruption, especially in humans or transgenic rodents that are APOE4 positive, through its effects on MMP-9 [20], [21]. CCI induced changes at 24 h in AQP-4, HIF-1, and both MMP-2 and -9. Changes were still present in comparison to shams at day 7 for HIF-1 and MMP-9. In contrast, the effect of CCI on Cyclophilin A was delayed, occurring at day 7 but without effect 24 h after CCI. We found that mHW counteracted the CCI-induced perturbance of each of these factors. These effects of mHW countering the CCI-induced alterations for AQP-4, HIF-1, the MMP’s, and Cyclophilin A provide mechanisms by which mHW can protect against edema and BBB disruption.

Alterations in AQP-4, which others have also found is down regulated in TBI, has been associated with alterations in clearance of Aβ through its effects on the circulation of interstitial fluid in the brain. We, however, found that neither the brain-to-blood clearance of Aβ nor the reabsorption of cerebrospinal fluid back into the circulation was altered by CCI at the time points examined here. Although CCI did affect protein levels of APP and the Aβ’s, mHW did not alter the effects of CCI.

Treatment with mHW totally blocked the pathological phosphorylated tau changes induced by CCI. In contrast, total tau was not altered at 12, 18, 24 or 48 h after CCI. TBI causes early transient accumulation of abnormally phosphorylated tau and repeated injury can result in chronic traumatic encephalopathy which is classified as a tauopathy characterized by tau hyperphosphorylation, and accumulation of conformationally altered or aggregated pathological tau. Pathological tau changes are also a hallmark of Alzheimer’s disease, ultimately resulting in neurofibrillary tangles, and is one of the findings that suggests TBI and Alzheimer’s disease may share pathophysiological mechanisms. Here, we found elevations of phosphorylated tau after CCI in both hippocampal and cortical regions as detected by AT8 and Alz50, but not in total tau. The AT8 antibody detects tau that is phosphorylated at Ser202 and Thr205, a phosphorylation pattern typical of the immature neurofibrillary tangles of Alzheimer’s disease. Alz50 also recognizes a pre-tangle phosphorylation pattern similar to one found in Alzheimer’s. Treatment with mHW totally blocked the detection of these CCI-induced forms of phosphorylated tau.

Neuroinflammation is thought to be a significant driver of the long-term pathology resulting from TBI. Specific agents of inflammation, particularly cytokines, likely play roles in both disease progression and recovery. Neuroinflammation is an early event in CCI as seen here by elevations in cytokines and cytokine-related genes. CCI-induced peripheral inflammation as assessed by serum cytokines was, with the exception of an early rise in serum G-CSF, unremarkable. The inflammatory drivers, then, are likely centered in the CNS rather than as peripheral-to-brain events. This is not surprising considering the CCI model of TBI inflicts significant damage directly to the CNS, although it is surprising given that neuroinflammation can influence the peripheral immune system. Immunohistochemical staining suggested that microglia, but not astrocytes, were activated by CCI. Staining showed that microglia were activated in both the cortex and hippocampus. The effect of mHW was profound on CCI-induced microglial activation and seemed to have reduced levels of activation lower than those seen in sham mice, especially in cortex. Of the 14 cytokines whose levels were increased by CCI, 7 of them had levels that were significantly affected by mHW at one or more time points. However, mHW mostly induced a further increase in brain cytokine levels, elevating them even higher than did CCI alone. This disconnect between the effect of mHW on microglial activation as detected by Iba1 staining and on brain cytokine levels suggests that another cell type is responsible for cytokine production. Both brain endothelial cells and pericytes are known to secrete cytokines constitutively and in response to neuroimmune stimuli [22], [23], [24]. Additionally, the transport rate of cytokines across the BBB could be increased as shown to occur in other types of CNS injury [25], [26], [27], [28] or CCI could increase immune cell trafficking into the CNS and their contribution to the pool of CNS cytokines [29]. The results further suggest, given the therapeutic effects of mHW, that the 7 cytokines which had further elevations with mHW treatment may play a therapeutic role in recovery from TBI rather than a pathological role in progression. Some cytokines, such as TNF [30], are notorious for being either beneficial or deleterious depending on concentration and context and TBI is one such condition in which cytokines play both of these roles [8].

Despite the paradoxic effect on brain cytokine protein levels, the effects of mHW was to reverse CCI-induced increases in the expression of genes related to cytokines. Treatment with mHW affected genes whose proteins were active at the cell surface and tended to not affect CCI-induced activated genes that affected intracellular, nuclear, or ATP and nucleotide processes. The surface genes affected were those that tended to relate to cytokine release and secretion, oxidation and carbohydrate metabolism, leukocyte trafficking, and receptors, channels, and transporters.

Recent work has shown that brain pericytes are critical to protecting the BBB and the neurovascular unit from oxidative stress, but are themselves very sensitive to oxidative stress, especially as induced by mitochondrial respiration [18], [31], [32]. The work summarized in Table 2 shows that mHW reverses the CCI-induced altered gene expression related to oxidation and carbohydrate metabolism and, as such, brain pericytes are the logical cell type to examine when assessing the protective effects of a CNS anti-oxidant. Assessment of cellular respiration showed that exposure of brain pericytes to molecular hydrogen for 24 h increased basal respiration, reserve capacity, and non-mitochondrial respiration, where “non-mitochondrial respiration” is defined as oxygen use that occurs despite shutdown of the electron transport chain (Figure 10). In cells not exposed to the metabolic stressors (Figure 10D), ATP production as assessed by oxygen consumption was not increased but direct measurement of ATP showed that it was, again indicating ATP production independent of oxygen use. In pericytes in which the electron transport chain was inactivated (Figure 11), molecular hydrogen was without effect on oxygen consumption or ECAR, but ATP levels were found to be elevated. Thus, the work in figures 10 and 11 provide evidence that molecular hydrogen can support ATP production independent of the electron transport chain. We propose that these findings can all be explained by the Jagendorf reaction, defined as ATP production that results from an inequality of hydrogen ion electrochemical activity that has been produced independently of the electron transport chain [33]. Under normal circumstances, the electron transport chain establishes the proton gradient and the gradient results in ATP production. Jagendorf, however, showed that the electron transport chain could be bypassed and ATP produced independently of it by establishing the hydrogen gradient with an acid environment. We propose that molecular hydrogen may also be producing a hydrogen gradient, thus promoting mitochondrial ATP production independent of electron transport chain activity. Thus, molecular hydrogen may be working in part by overcoming the deficit in ATP that cells undergoing TBI, and so having mitochondrial damage [34], experience.

Overall, these studies provide proof of principle that mHW can potently reverse, block, or attenuate many of the effects of CCI. These include effects on edema formation, tau pathology, regulators of fluid and BBB functions, neuroinflammation, and gene expression. These studies were limited in that they did not evaluate mHW treatment without a pretreatment phase, but suggest that mHW could be an important, nontoxic treatment for acute TBI. Because of its nontoxicity and ease of administration, mHW could be readily adapted for clinical, emergency, and even first responder use.

## References

[1] Sayer NA (2012) Traumatic brain injury and its neuropsychiatric sequelae in war veterans. Annu Rev Med 63: 405–419. 22248327 10.1146/annurev-med-061610-154046

[2] Wick JY (2012) Traumatic brain injury: special problem, special care. Consult Pharm 27: 392–399. 22698546 10.4140/TCP.n.2012.392

[3] Cernak I (2005) Animal models of head trauma. NeruoRx 2: 410–422. 10.1602/neurorx.2.3.410 PMC1144485 16389305

[4] Bazarian JJ, Cernak I, Noble-Haeusslein L, Potolicchio S, Temkin N (2009) Long-term neurologic outcomes after traumatic brain injury. J Head Trauma Rehabil 24: 439–451. 19940677 10.1097/HTR.0b013e3181c15600

[5] Deng Y, Thompson BM, GAo X, Hall ED (2007) Temporal relationship of peroxynitrite-induced oxidative damage, calpain-mediated cytoskeletal degradation and neurodegeneration after traumatic brain injury. Experimental Neurology 205: 154–165. 17349624 10.1016/j.expneurol.2007.01.023 PMC1950332

[6] Hawkins BE, Krishnamurthy S, Castillo-Carranza DL, Sengupta U, Prough DS, et al.. (2013) Rapid Accumulation of Endogenous Tau Oligomers in a Rat Model of Traumatic Brain Injury: Possible Link Between TBI and Sporadic Tauopathies. J Biol Chem. 10.1074/jbc.M113.472746 PMC3675635 23632019

[7] Johnson VE, Stewart W, Smith DH (2012) Widespread tau and amyloid-beta pathology many years after a single traumatic brain injury in humans. Brain Pathol 22: 142–149. 21714827 10.1111/j.1750-3639.2011.00513.x PMC3979351

[8] Morganti-Kossman MC, Lenzlinger PM, Hans V, Stahel P, Csuka E, et al (1997) Production of cytokines following brain injury: beneficial and deleterious for the damaged tissue. Molecular Psychiatry 2: 133–136. 9106236 10.1038/sj.mp.4000227

[9] Ohsawa I, Ishikawa M, Takahashi K, Watanabe M, Nishimaki K, et al (2007) Hydrogen acts as a therapeutic antioxidant by selectively reducing cytotoxic oxygen radicals. Nature Medicine 13: 688–694. 10.1038/nm1577 17486089

[10] Miyamoto K, Tsumuraya T, Ohtaki H, Dohi K, Satoh K, et al.. (2014) PACAP38 suppresses cortical damage in mice with traumatic brain injury by enhancing antioxidant activity. J Mol Neurosci epub. 10.1007/s12031-014-0309-4 24907941

[11] Keep RF, Hua Y, Xi G (2012) Brain water content. A misunderstood measurement? Transl Stroke Res 3: 263–265. 22888371 10.1007/s12975-012-0152-2 PMC3413327

[12] Ishihara T, Hong M, Zhang B, Nakagawa Y, Lee MK, et al (1999) Age-dependent emergence and progression of a tauopathy in transgenic mice overexpressing the shortest human tau isoform. Neuron 24: 751–762. 10595524 10.1016/s0896-6273(00)81127-7

[13] Guthrie CR, Schellenberg GD, Kraemer BC (2009) SUT-2 potentiates tau-induced neurotoxicity in Caenorhabditis elegans. Hum Mol Genet 18: 1825–1838. 19273536 10.1093/hmg/ddp099 PMC2722225

[14] Erickson MA, Niehoff ML, Farr SA, Morley JE, Dillman LA, et al (2012) Peripheral administration of antisense oligonucleotides targeting the amyloid beta protein precursor reverses ABPP and LRP-1 overexpression in aged SAMP8 mouse brain. J Alzheimer’s Dis 28: 951–960. 22179572 10.3233/JAD-2011-111517

[15] Silverberg GD, Messier AA, Miller MC, Machan JT, Majmudar SS, et al (2010) Amyloid efflux transporter expression at the blood-brain barrier declines in normal aging. J Neuropathol Exp Neurol 69: 1034–1043. 20838242 10.1097/NEN.0b013e3181f46e25

[16] De Gasperi R, Gama Sosa MA, Kim SH, Steele JW, Shaughness MC, et al (2012) Acute blast injury reduces brain abeta in two rodent species. Front Neurol 3: 177. 23267342 10.3389/fneur.2012.00177 PMC3527696

[17] Banks WA, Fasold MB, Kastin AJ (1997) Measurement of efflux rate from brain to blood. In: Irvine GB, Williams CH, editors. Methods in Molecular Biology: Neuropeptides Protocols. Totowa, NJ: Humana Press. 353–360. 10.1385/0-89603-399-6:353 9031222

[18] Shah GN, Price TO, Banks WA, Morofuji Y, Kovac A, et al (2012) Pharmacological inhibition of mitochondrial carbonic anhydrases protects mouse cerebral pericytes from high glucose-induced oxidative stress and apoptosis. J Pharmacol Exp Therap 344: 637–645. 23249625 10.1124/jpet.112.201400 PMC3583505

[19] Higashida T, Kreipke CW, Rafols JA, Peng C, Schafer S, et al (2011) The role of hypoxia-inducible factor-1alpha, aquaporin-4, and matrix metalloproteinase-9 in blood-brain barrier disruption and brain edema after traumatic brain injury. J Neurosurg 114: 92–101. 20617879 10.3171/2010.6.JNS10207

[20] Bell RD, Winkler EA, Singh I, Sagare AP, Deane R, et al (2012) Apolipoprotein E controls cerebrovascular integrity via cyclophilin A. Nature. 485: 512–516. 10.1038/nature11087 PMC4047116 22622580

[21] Halliday MR, Pomara N, Sagare AP, Mack WJ, Frangione B, et al (2013) Relationship between cyclophilin A levels and matrix metalloproteinase 9 activity in cerebrospinal fluid of cognitively normal apolipoprotein E4 carriers and blood-brain barrier breakdown. JAMA 70: 1198–1200. 10.1001/jamaneurol.2013.3841 PMC4047029 24030206

[22] Reyes TM, Fabry Z, Coe CL (1999) Brain endothelial cell production of a neuroprotective cytokine, interleukin-6, in response to noxious stimuli. Brain Research 851: 215–220. 10642846 10.1016/s0006-8993(99)02189-7

[23] Verma S, Nakaoke R, Dohgu S, Banks WA (2006) Release of cytokines by brain endothelial cells: a polarized response to lipopolysaccharide. Brain, Behavior, and Immunity 20: 449–455. 10.1016/j.bbi.2005.10.005 16309883

[24] Kovac A, Erickson MA, Banks WA (2011) Brain microvascular pericytes are immunoactive in culture: cytokine, chemokine, nitric oxide, and LRP-1 expression in response to lipopolysaccharide. Journal of Neuroinflammation 8: 139. 21995440 10.1186/1742-2094-8-139 PMC3207972

[25] Pan W, Banks WA, Kennedy MK, Gutierrez EG, Kastin AJ (1996) Differential permeability of the BBB in acute EAE: enhanced transport of TNF-alpha. American Journal of Physiology 271: E636–E642. 8897850 10.1152/ajpendo.1996.271.4.E636

[26] Pan W, Kastin AJ (2001) Increase in TNF alpha transport after SCI is specific for time, region, and type of lesion. Experimental Neurology 170: 357–363. 11476601 10.1006/exnr.2001.7702

[27] Pan W, Banks WA, Kastin AJ (1998) Permeability of the blood-brain barrier to neurotrophins. Brain Research 788: 87–94. 9554964 10.1016/s0006-8993(97)01525-4

[28] Pan W, Cain C, Yu Y, Kastin AJ (2006) Receptor-mediated transport of LIF across blood-spinal cord barrier is upregulated after spinal cord injury. Journal of Neuroimmunology 174: 119–125. 16563523 10.1016/j.jneuroim.2006.02.006

[29] JIn X, Ishii H, Bai Z, Itokazu T, Yamashita T (2012) Temporal changes in cell marker expression and cellular infliltration in a controlled cortical impact model in adult male C57BL/6 mice. PLoS One 7: e41892. 22911864 10.1371/journal.pone.0041892 PMC3404031

[30] Pan W, Zadina JE, Harlan RE, Weber JT, Banks WA, et al (1997) Tumor necrosis factor-alpha: a neuromodulator in the CNS. Neuroscience and Biobehavioral Reviews 21: 603–613. 9353794 10.1016/s0149-7634(96)00047-4

[31] Price TO, Eranki V, Banks WA, Ercal N, Shah GN (2012) Topiramate treatment protects blood-brain barrier pericytes from hyperglycemia-induced oxidative damage in diabetic mice. Endocrinology 153: 362–372. 22109883 10.1210/en.2011-1638 PMC3249670

[32] Shah GN, Price TO, Banks WA, Morofuji Y, Kovac A, et al (2013) Pharmacological inhibition of mitochondrial carbonic anhydrases protects mouse cerebral pericytes from high glucose-induced oxidative stress and apoptosis. J Pharmacol Exp Therap 344: 637–645. 23249625 10.1124/jpet.112.201400 PMC3583505

[33] Jagendorf AT, Uribe E (1966) ATP formation caused by acid-base transition of spinach chloroplasts. Proc Natl Acad Sci USA 55: 170–177. 5220864 10.1073/pnas.55.1.170 PMC285771

[34] Soustiel JF, Larisch S (2010) Mitochondrial damage: a target for new therapeutic horizons. Neurotherapeutics 7: 13–21. 20129493 10.1016/j.nurt.2009.11.001 PMC5084108

